# Interplay Between Aging and Glial Cell Dysfunction: Implications for CNS Health

**DOI:** 10.3390/life15101498

**Published:** 2025-09-23

**Authors:** Mario García-Domínguez

**Affiliations:** 1 Program of Immunology and Immunotherapy, CIMA-Universidad de Navarra, 31008 Pamplona, Spain; mgdom@unav.es; 2 Department of Immunology and Immunotherapy, Clínica Universidad de Navarra, 31008 Pamplona, Spain; 3 Centro de Investigación Biomédica en Red de Cáncer (CIBERONC), 28029 Madrid, Spain

**Keywords:** aging, glial cells, astrocytes, microglia, oligodendrocytes, neuroinflammation, inflammaging, senescence

## Abstract

Aging is accompanied by complex cellular and molecular changes that compromise CNS function. Among these, glial cells (astrocytes, microglia, and oligodendrocytes) play a central role in maintaining neural homeostasis, modulating synaptic activity, and supporting metabolic demands. Emerging evidence indicates that aging disrupts glial cell physiology through processes including mitochondrial dysfunction, impaired proteostasis, chronic low-grade inflammation, and altered intercellular signaling. These alterations contribute to synaptic decline, myelin degeneration, and persistent, low-grade inflammation of the CNS. This review synthesizes current knowledge on the bidirectional relationship between aging and glial cell dysfunction, highlighting how age-related systemic and CNS-specific factors exacerbate glial impairments and, in turn, accelerate neural deterioration. Finally, this study discusses some potential therapeutic strategies aimed at preserving or restoring glial function to promote CNS resilience in aging populations. Understanding this interplay offers critical opportunities for mitigating cognitive decline and improving quality of life in older adults.

## 1. Introduction

Aging is a multifactorial biological process characterized by a progressive decline in physiological integrity, reduced cellular resilience, and increased susceptibility to disease [1,2,3]. Within the CNS, aging not only affects neurons but also induces profound alterations in the function and homeostatic regulation of glial cells, including astrocytes, microglia, and oligodendrocytes [4]. These non-neuronal cell populations are now recognized as dynamic regulators of synaptic plasticity, neurovascular coupling, metabolic homeostasis, and neuroimmune surveillance [5,6]. The cumulative molecular and cellular alterations that accompany aging perturb these glial functions, thereby accelerating cognitive decline, increasing vulnerability to neurodegenerative disorders, and undermining brain health [7,8].

At the molecular level, aging induces extensive reprogramming of glial cell gene expression, driven by the cumulative impact of epigenetic drift (defined as stochastic alterations in the epigenome that accumulate over time) encompassing changes in DNA methylation patterns, histone modifications, and chromatin remodeling [9,10,11]. In aging glial cells, chromatin accessibility is often reduced at loci associated with neuroprotective and metabolic genes, while pro-inflammatory and stress-response genes might become more accessible, driving a maladaptive transcriptional shift [12,13]. Mitochondrial dysfunction, a well-established hallmark of aging, plays a central role in this process [14]. In glial cells, compromised electron transport chain efficiency reduces ATP production, impairing the high-energy-demanding functions of those cells [15]. This inefficiency also leads to excessive production of ROS, which induce oxidative damage on lipids, proteins, and nucleic acids [15].

Astrocytes, which play essential roles in maintaining CNS homeostasis, supporting neuronal function, and regulating the BBB [16], undergo a shift toward a reactive phenotype in response to aforementioned insults. Their reactive state is characterized by hypertrophy, increased expression of intermediate filament proteins like GFAP and vimentin, and the secretion of several pro-inflammatory mediators, such as IL-1β, TNF-α, and CCL2 [17,18]. Sustained activation of the NF-κB signaling pathway locks astrocytes into an inflammatory state, further impairing their neuroprotective roles [19]. One functional consequence is the reduction in glutamate clearance due to decreased expression of excitatory amino acid transporters EAAT1 and EAAT2, creating conditions favorable for excitotoxic neuronal damage [20].

Microglia, the resident immune sentinels of the CNS [21], undergo a parallel but distinct aging trajectory, a process often known as microglial priming [22]. With aging process, PRR pathways, particularly TLR4 signaling, become dysregulated, making microglia hyperresponsive to secondary insults including infections or trauma [23]. Primed microglia exhibit amplified and sustained inflammatory responses, but paradoxically show reduced phagocytic efficiency, compromising the clearance of myelin debris, apoptotic cells, and aggregated proteins such as amyloid-β [24,25,26]. Dysfunction in purinergic signaling, especially through P2X7 and P2Y12 receptors, further disrupts microglial chemotaxis and injury sensing [27,28]. Autophagic flux declines with age, leading to lysosomal dysfunction, which traps damaged organelles and undigested materials inside the cell [29]. This failure of clearance mechanisms sustains the presence of DAMPs in the CNS microenvironment, perpetuating a self-reinforcing cycle of inflammation and neuronal stress [30].

OPCs, the main source of new myelinating oligodendrocytes in the adult CNS [31], also exhibit significant age-related decline [32]. Aging OPCs show impaired proliferation and differentiation capacity, largely driven by epigenetic repression of the genes implied in myelin synthesis, such as MBP and PLP1 [33]. Furthermore, OPCs become less responsive to mitogenic growth factors, including PDGF-A and FGF2, which usually promote OPC expansion and maturation [34,35]. The loss of regenerative capacity impairs remyelination efficiency and contributes to the progressive degradation of white matter integrity, a crucial substrate for cognitive processing speed and executive function [36].

These issues are exacerbated by systemic aging factors, including chronic low-grade inflammation (known as inflammaging), characterized by increased levels of circulating pro-inflammatory cytokines, as well as alterations in metabolic hormones such as insulin and IGF-1 [37,38]. These systemic molecules facilitate glial senescence via activation of the cell cycle inhibitors p16 and p21, inducing an irreversible growth arrest that further impairs the CNS reparative and adaptive capacity [39]. Over time, these converging cellular and molecular deficits create a CNS environment more susceptible to neurodegenerative processes [40]. Furthermore, these glial modifications do not occur in isolation but rather within a complex and bidirectional interplay with aging neurons, vascular elements, and the immune system [41]. The concept of the Neuro-Glia-Vascular unit underscores the key role of glial cells in the regulation of CNS homeostasis, whereby age-related dysfunctions in a single cell type can propagate disturbances throughout the neural network [42]. Such perturbations can impair synaptic integrity and neurovascular coupling, ultimately contributing to and neurodegenerative processes [43].

Finally, this article offers an extensive analysis of glial cells and their involvement in the aging process, beginning with the cellular and molecular modifications that glial cells undergo during the aging process. Subsequent sections address the impact of these alterations on brain health, highlighting their contribution to cognitive deterioration. Finally, this article explores preventive and therapeutic strategies aimed at restoring glial function and enhancing neural resilience during brain aging.

## 2. Aging-Associated Changes in Glial Cells

Glial cells undergo progressive structural and functional modifications during aging that can profoundly influence CNS homeostasis. These changes are not merely secondary to neuronal decline but represent intrinsic alterations in glial biology, encompassing shifts in morphology, signaling dynamics, metabolic activity, and immune responsiveness. The following sections summarize the principal morphological and physiological alterations identified in glial populations during the aging process.

### 2.1. Morphological Alterations

During the aging process, glial cells orchestrate numerous morphological alterations driven by the interplay between cell-autonomous senescence-associated molecular mechanisms and the cumulative impact of chronic, low-grade neuroinflammatory stimuli sustained throughout the lifespan within the CNS microenvironment [44].

**Figure 1:**
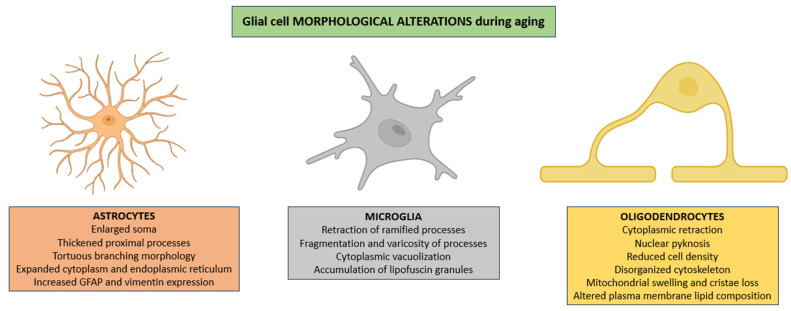
Morphological alterations in glial populations across the aging process. In aged CNS, astrocytes show pronounced hypertrophy, irregular branching, cytoplasmic enlargement, and increased GFAP and vimentin expression, reflecting a chronic reactive state. Microglia display progressive dystrophic features, including process retraction and fragmentation, cytoplasmic vacuolization, and lipofuscin deposition. Oligodendrocytes show cytoplasmic shrinkage, nuclear pyknosis, reduced cell density, cytoskeletal disruption, mitochondrial structural defects, and altered plasma membrane lipid composition, collectively impairing white matter integrity and glial–neuronal signaling. Abbreviations: GFAP (glial fibrillary acidic protein).

Astrocytes frequently exhibit pronounced hypertrophy characterized not only by enlargement of the soma and thickening of proximal processes but also by a notable increase in cytoplasmic volume, an expansion of the endoplasmic reticulum, and a redistribution of intermediate filaments that results in tortuous branching morphologies [45]. All of these changes are usually accompanied by enhanced expression of cytoskeletal proteins such as GFAP and vimentin, reflecting a shift toward a chronically reactive state that alters astrocytic interactions with both neuronal and vascular elements (Figure 1) [46].

In parallel, microglia exhibit progressive dystrophic changes characterized by retraction of ramified processes, which are essential for environmental surveillance; fragmentation and varicosity of the remaining processes, consistent with cytoskeletal disintegration; cytoplasmic vacuolization, suggestive of impaired autophagic flux; and intracellular accumulation of autofluorescent lipofuscin granules, representing morphological correlates of diminished lysosomal degradative capacity (Figure 1) [47,48,49].

Oligodendrocytes frequently exhibit cytoplasmic retraction, nuclear pyknosis reflecting chromatin condensation, and a marked reduction in cellular density within affected white matter regions [50]. These alterations promote the age-related thinning of the myelin sheath, patchy demyelination, and the disruption of axonal conduction fidelity, ultimately compromising the integrity of white matter tracts [51,52]. These modifications are frequently accompanied by profound disturbances in cytoskeletal architecture, including disorganization of intermediate filaments, disruption of actin filaments, and destabilization of microtubules, which together undermine intracellular transport and process stability [53,54]. Additionally, mitochondrial structural derangements (such as swelling, loss of cristae definition, and outer membrane rupture) disrupt bioenergetic homeostasis and exacerbate oxidative stress [55]. Finally, alterations in plasma membrane lipid composition, particularly reductions in polyunsaturated fatty acids and cholesterol distribution, further impair membrane fluidity, receptor clustering, and ion channel function, thus compounding deficits in glial–neuronal communication and metabolic support (Figure 1) [56].

### 2.2. Physiopathological Alterations

In the aging CNS, glial cells exhibit progressive dysfunction arising from interrelated pathological processes, like bioenergetic impairment due to mitochondrial dysfunction and oxidative stress, disrupted neuroglial communication, defective proteostasis and autophagy, diminished regenerative potential, and cellular senescence, collectively fostering neuroinflammation and heightened neural vulnerability.

#### 2.2.1. Mitochondrial Dysfunction and Oxidative Stress

Mitochondrial dysfunction constitutes a critical driver of glial cell impairment within the aging CNS, where it is linked to the progressive accumulation of oxidative stress [57]. With aging, mitochondrial quality control mechanisms (PINK1/Parkin-mediated mitophagy) demonstrate reduced efficiency, enabling the persistence and accumulation of bioenergetically compromised mitochondria [58]. This is aggravated by an imbalance between ROS production and the antioxidant defense mechanisms of the CNS, as evidenced by the age-related downregulation of mitochondrial SOD2 activity [59].

In astrocytes, aging compromises mitochondrial electron transport chain efficiency, predominantly at complexes I (NADH:ubiquinone oxidoreductase) and IV (cytochrome c oxidase), leading to reduced ATP synthesis and increased electron leakage that increases reactive oxygen species production [60]. This leakage encourages excessive generation of ROS, such as superoxide anions (O_2_^−^) and hydrogen peroxide (H_2_O_2_) [61,62]. Elevated ROS levels drive the oxidation of membrane lipids [63], structural and enzymatic proteins [64], and nucleic acids [65], thereby disrupting astrocytic metabolic homeostasis and activating redox-sensitive transcription factors (like NF-κB), which contribute to neuroinflammatory signaling (Table 1) [66].

In microglia, mitochondrial respiratory dysfunction impairs oxidative phosphorylation (OXPHOS) capacity, inducing a compensatory metabolic shift toward aerobic glycolysis [67,68]. This metabolic shift, while sustaining basal energy levels, drives a prolonged pro-inflammatory phenotype (commonly identified as “M1-like” activation) and compromises microglial phagocytic clearance of cellular debris, protein aggregates, and apoptotic cells [69]. The persistence of this dysfunctional metabolic state exacerbates neuroinflammation and reduces the capacity for resolution of CNS injury (Table 1) [70].

Finally, oligodendrocytes, which depend extensively on mitochondrial ATP production for the synthesis and maintenance of myelin sheaths [71], are particularly susceptible to oxidative damage (targeting mtDNA) and to lipid peroxidation within myelin membranes, a process intensified by the high content of polyunsaturated fatty acids (Table 1) [55,72].

#### 2.2.2. Impairment of Proteostasis and Autophagy

The impairment of proteostasis and autophagy in glial cells is a pivotal factor in the aging CNS, contributing significantly to neurodegeneration and cognitive decline [73,74]. Proteostasis, encompassing the intricate regulation of protein synthesis, folding, trafficking, and degradation, becomes increasingly compromised in glial cells, due to cumulative oxidative damage, mitochondrial dysfunction, and neuroinflammatory signaling characteristic of aging [75,76]. In contrast, autophagy, a conserved catabolic process responsible for the degradation and recycling of damaged organelles, protein aggregates, and other cytoplasmic components, is impaired in glial cells during the aging process [75,77].

In astroglia, the disruption of proteostasis manifests as impaired function of molecular chaperones including heat shock proteins (e.g., Hsp70), resulting in the accumulation of misfolded proteins and cytotoxic aggregates [78]. This proteotoxic stress interferes with regulation of glutamate and ion homeostasis, exacerbating neuronal excitotoxicity [79]. Furthermore, aging astrocytes exhibit a marked reduction in autophagic flux, evidenced by decreased expression of crucial autophagy-related genes such as Beclin-1 and LC3 [80], alongside lysosomal dysfunction characterized by impaired acidification and enzymatic activity, which impairs the removal of dysfunctional mitochondria and protein aggregates (Table 1) [81].

In microglia, proteasomal insufficiency combined with defective autophagy leads to intracellular accumulation of dysfunctional mitochondria and protein inclusions, which trigger sustained inflammasome activation (e.g., NLRP3) and release of some pro-inflammatory cytokines (e.g., IL-1β and TNF-α) [82,83,84]. This neuroinflammatory milieu further disrupts proteostasis by increasing oxidative damage and impairing chaperone-mediated autophagy (CMA), a selective pathway crucial for degradation of cytosolic proteins containing KFERQ-like motifs (Table 1) [85].

Oligodendrocytes experience pronounced impairments in proteostasis and autophagy as a consequence of aging [86,87]. Impaired UPS and autophagic clearance of myelin debris and damaged organelles compromises oligodendrocyte viability and myelin integrity, leading to demyelination and reduced nerve conduction [88,89]. Moreover, inhibition of the proteasome during aging induces DNA fragmentation and promotes oligodendrocyte degeneration (Table 1) [90].

#### 2.2.3. Cellular Senescence

Cellular senescence in glial cells represents a key contributor to age-associated neurodegenerative decline, orchestrated via molecular networks that integrate DNA damage responses, epigenetic remodeling, and inflammatory signaling [91,92].

In astrocytes, chronic exposure to oxidative stress and mitochondrial dysfunction underlies the accumulation of both nuclear and mtDNA lesions, specifically double-strand breaks (DSBs) and oxidative base modifications such as 8-oxoguanine, which activate the DNA damage response (DDR) through ATM and ATR kinases [93,94,95]. These pathological processes phosphorylate downstream CHK1 and CHK2 effectors, stabilizing p53, which transcriptionally upregulates p21, inducing a stable cell cycle arrest at the G1 to S phase transition [96]. Parallel induction of the INK4a/ARF locus enhances p16 expression, which suppresses CDK4/6 kinase activity, thereby inhibiting phosphorylation of the retinoblastoma protein (Rb), resulting in the growth-suppressive conformation and enforcing a stable senescent cell cycle arrest [97]. These checkpoints converge to establish a robust proliferative arrest in astrocytes, thereby preventing uncontrolled cellular proliferation while simultaneously compromising their neurotrophic support functions [98]. Senescent astrocytes also develop a senescence-associated secretory phenotype (SASP), orchestrated at the transcriptional level by NF-κB [99,100]. This SASP includes the production and secretion of some pro-inflammatory cytokines (e.g., IL-6 and IL-8), chemokines (e.g., CXCL10), growth factors (e.g., TGF-β), and matrix metalloproteinases (e.g., MMP-3 and MMP-9) [101,102]. These factors modulate the microenvironment, disrupting BBB integrity, inducing astrogliosis, and impairing synaptic plasticity [103]. Epigenetic remodeling in astrocytes, defined by the accumulation of methylation marks like H3K4me3 and the loss of histone acetylation marks such as H3K27ac, reinforces SASP gene expression and stabilizes the senescent phenotype [104,105]. Finally, DNA methylation changes (e.g., hypermethylation of neuroprotective gene promoters alongside global hypomethylation) alter chromatin accessibility, further strengthening dysfunctional phenotypes (Table 1) [106].

In microglia, senescence is driven by telomere attrition, oxidative stress, and persistent low-grade inflammatory stimuli [107]. Telomere attrition activates DDR signaling (through ATM and ATR kinases), upregulating p16, p21 and p53, thereby strengthening cell cycle arrest [108]. Moreover, senescent microglia exhibit a pronounced pro-inflammatory SASP, with increased secretion of several cytokines (e.g., IL-1β, TNF-α, and IFN-γ), chemokines (e.g., CCL5 and CXCL10), and increased NO production [109,110]. Epigenetic alterations, such as increased expression of some HDACs (e.g., HDAC1) sustain transcriptional activation of the SASP microglia [111]. Finally, increased DNA demethylation patterns have been observed in the aged CNS, thereby contributing to a heightened activation of pro-inflammatory pathways (Table 1) [112].

OPCs and mature oligodendrocytes display unique senescence-associated alterations during aging [113]. In response to aging-induced demyelination, the p53/p21 and p16/Rb pathways are robustly activated [114]. On the other hand, senescent OPCs adopt an SASP phenotype that includes secretion of matrix metalloproteinases (e.g., MMP-3) and several pro-inflammatory cytokines (e.g., IL-6 and TNF-α), disrupting extracellular matrix integrity and impeding neuron-glia signaling, key process for remyelination [32,115]. Finally, during the aging process, a reduction in HDAC activity (e.g., HDAC1, HDAC2, HDAC3, and HDAC8) is observed, and a strong decrease in global DNA hypomethylation in aged oligodendrocytes correlates with reduced DNMT1 expression (Table 1) [116,117].

#### 2.2.4. Alterations in Intercellular Signaling and Communication

Aging drives several profound changes in intercellular signaling and communication within the CNS, impacting glial cell function [118]. These alterations involve cellular, molecular, and network-scale modifications that perturb the precisely regulated crosstalk between neurons and glial cells, as well as interglial communication among glial subpopulations [119,120]. Such dysregulation of glial signaling pathways undermines the capacity of glial cells to maintain homeostasis, modulate synaptic activity, and orchestrate immune responses [121]. Consequently, these signaling perturbations contribute to the progressive decline in neural plasticity and cognitive function observed during aging [122,123].

Astrocytes, which normally support neuronal activity via Ca^2+^-dependent gliotransmitter release and regulation of extracellular neurotransmitter levels [124], exhibit dysregulated intracellular Ca^2+^ dynamics and reduced expression of connexins (e.g., Cx43), leading to impaired gap junctional communication [125]. This disruption impairs their ability to adequately propagate Ca^2+^ waves and release gliotransmitters (e.g., ATP and D-serine), essential modulators of synaptic transmission (Table 1) [126,127].

Conversely, the composition and molecular cargo of extracellular vesicles (EVs) released by aged microglia exhibit substantial alterations that markedly modulate intercellular signaling within the CNS [128]. These EVs, which include exosomes and microvesicles, serve as key mediators of cell-to-cell communication by transferring bioactive molecules such as proteins, lipids, and miRNAs to recipient neurons and glial cells [129]. In the context of aging, microglia-derived EVs show a modified proteomic and lipidomic profile characterized by increased contents of several pro-inflammatory cytokines (e.g., IL-1β and TNF-α), DAMPs, lipid peroxidation products, and miRNAs that regulate inflammatory pathways and immune responses (e.g., miR-192; Table 1) [130,131,132,133,134].

Finally, in the aging CNS, the accumulation of myelin debris resulting from impaired clearance mechanisms represents a key pathological feature that profoundly impacts oligodendrocyte function and remyelination capacity [135]. This impairment in the clearance of myelin debris promotes the recruitment and activation of peripheral and CNS resident inflammatory cells, accompanied by sustained microglial activation (Table 1) [136,137].

**Table 1:** Table showing age-related alterations in glial cell function. Abbreviations: ATP (adenosine triphosphate), ROS (reactive oxygen species), CNS (central nervous system), mtDNA (mitochondrial DNA), DDR (DNA damage response), ATM (ataxia-telangiectasia mutated), ATR (ATM and Rad3-related), p16 (cyclin-dependent kinase inhibitor 2A), CDK4/6 (cyclin-dependent kinases 4 and 6), Rb (retinoblastoma protein), SASP (senescence-associated secretory phenotype), NF-κB (nuclear factor kappa-light-chain-enhancer of activated B cells), H3K4me3 (histone 3 lysine 4 trimethylation), H3K27ac (histone H3 lysine 27 acetylation), IL-1β (interleukin 1 beta), TNF-α (tumor necrosis factor alpha), IFN-γ (interferon gamma), CCL5 (C-C motif chemokine ligand 5), CXCL10 (C-X-C motif chemokine ligand 10), NO (nitric oxide), DNA (deoxyribonucleic acid), p53 (tumor protein p53), p21 (cyclin-dependent kinase inhibitor 1A), MMP-3 (matrix metalloproteinase 3), IL-6 (interleukin 6), ECM (extracellular matrix), HDAC (histone deacetylase), DNMT1 (DNA methyltransferase 1), Ca^2+^ (calcium ion), EV (extracellular vesicle), DAMP (damage-associated molecular pattern), and miRNA (microRNA).

Physiological Alteration	Cell Type	Consequences	References
Mitochondrial dysfunction and oxidative stress	Astroglia	Reduced efficiency of the mitochondrial electron transport chain Reduced ATP synthesis Enhanced electron leakage leading to elevated ROS production Oxidative damage to lipids, proteins, and nucleic acids Disruption of astrocytic metabolic homeostasis Activation of redox-sensitive transcription factors Amplification of neuroinflammatory signaling	[60,61,62,63,64,65,66]
	Microglia	Impaired oxidative phosphorylation capacity Compensatory metabolic shift toward aerobic glycolysis Sustained pro-inflammatory phenotype Reduced phagocytic clearance of cellular debris Exacerbation of neuroinflammation Impaired resolution of CNS injury	[67,68,69,70]
	Oligodendroglia	High susceptibility to oxidative damage and lipid peroxidation mtDNA disruption Compromised synthesis and maintenance of myelin sheaths	[55,71,72]
Impairment of proteostasis and autophagy	Astroglia	Impaired function of chaperones Accumulation of misfolded proteins and cytotoxic aggregates Disruption of glutamate and ion homeostasis Reduced autophagic flux Lysosomal dysfunction Impaired clearance of protein aggregates	[78,79,80,81]
	Microglia	Proteasomal insufficiency combined with defective autophagy Accumulation of dysfunctional mitochondria and protein inclusions Sustained inflammasome activation Release of pro-inflammatory cytokines Neuroinflammatory milieu exacerbates oxidative damage Impairment of chaperone-mediated autophagy	[82,83,84,85]
	Oligodendroglia	Pronounced impairments in proteostasis and autophagy Impaired proteolytic and autophagic degradation pathways Reduced oligodendrocyte viability and compromised myelin integrity Proteasome inhibition promotes oligodendrocyte degeneration	[86,87,88,89,90]
Cellular senescence	Astroglia	Accumulation of nuclear and mtDNA lesions Activation of DDR via ATM/ATR → stable G1/S arrest Activation of the p16-CDK4/6-Rb pathway reinforces G1/S arrest Development of SASP phenotype driven by NF-κB Epigenetic remodeling: accumulation of H3K4me3, loss of H3K27ac, promoter hypermethylation of neuroprotective genes, and global hypomethylation → stabilization of the SASP phenotype	[93,94,95,96,97,98,99,100,101,102,103,104,105,106]
	Microglia	SASP induced by telomere attrition and oxidative stress, among others DDR activation via ATM/ATR → proliferative arrest Pro-inflammatory SASP with secretion of IL-1β, TNF-α, IFN-γ, CCL5, and CXCL10, along with increased NO production Epigenetic alterations → increased SASP phenotype Age-associated DNA demethylation patterns amplify pro-inflammatory gene activation	[107,108,109,110,111,112]
	Oligodendroglia	Activation of p53/p21 and p16/Rb pathways in response to demyelination SASP phenotype with secretion of MMP-3, IL-6, and TNF-α → ECM disruption and impaired neuron-glia signaling Epigenetic changes: reduced HDAC activity and global DNA hypomethylation associated with decreased DNMT1 expression → compromised oligodendrocyte function	[113,114,115,116,117]
Alterations in intercellular signaling and communication	Astroglia	Dysregulated intracellular Ca^2+^ dynamics Reduced connexins expression Impaired gap junctional communication Deficient propagation of Ca^2+^ waves Reduced gliotransmitter release (e.g., ATP, D-serine) Disruption of synaptic transmission modulation	[124,125,126,127]
	Microglia	Altered composition and molecular cargo of EVs Modified proteomic and lipidomic profiles Increased content of pro-inflammatory cytokines and DAMPs Dysregulated transfer of regulatory miRNAs Exacerbation of inflammatory signaling and immune responses	[128,129,130,131,132,133,134]
	Oligodendroglia	Accumulation of uncleared myelin debris Impaired oligodendrocyte function Reduced remyelination capacity Activation of peripheral and CNS-resident inflammatory cells Sustained microglial activation	[135,136,137]

## 3. Consequences for CNS Health

These age-related glial alterations compromise the efficiency of neuron–glia metabolic coupling and reduce the fidelity of intercellular signaling pathways, ultimately undermining neuronal plasticity [138]. Maladaptive glial remodeling during aging drives neurodegeneration by chronic neuroinflammation, promoting synaptic dysfunction, facilitating myelin degradation, and increasing susceptibility to neurodegenerative diseases.

### 3.1. Synaptic Dysfunction

Synaptic dysfunction and cognitive decline are central features of brain aging, closely linked to both neuronal and glial alterations [139,140]. Glial cells, usually considered supportive, play key roles in maintaining CNS homeostasis and regulating synaptic plasticity, neurotransmitter clearance, and circuit-level signaling, fundamental processes for learning, memory, and executive functions [8,141].

**Figure 2:**
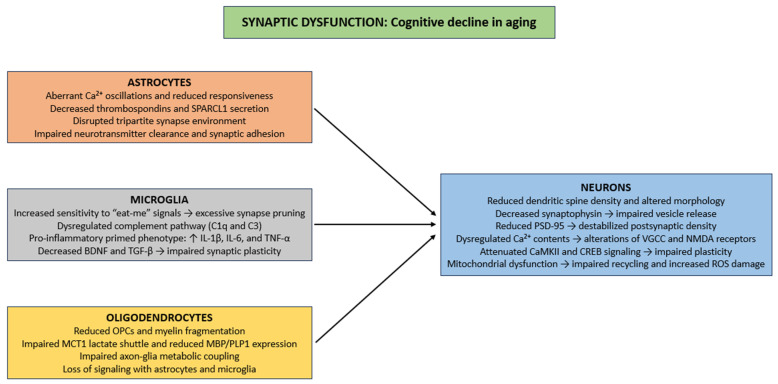
Cellular mechanisms of synaptic dysfunction in CNS aging. Abbreviations: Ca^2+^ (calcium ion), SPARCL1 (secreted protein acidic and rich in cysteine-like 1), C1q (complement component 1q), C3 (complement component 3), IL-1β (interleukin 1 beta), IL-6 (Interleukin 6), TNF-α (tumor necrosis factor alpha), BDNF (brain-derived neurotrophic factor), TGF-β (Transforming growth factor beta), OPC (oligodendrocyte precursor cell), MCT1 (monocarboxylate transporter 1), MBP (myelin basic protein), PLP1 (proteolipid protein 1), PSD-95 (postsynaptic density protein 95), VGCC (voltage-gated calcium channel), NMDA (N-methyl-D-aspartate), CaMKII (calcium/calmodulin-dependent protein kinase II), CREB (cAMP response element-binding protein), and ROS (reactive oxygen species).

Aging induces several structural and functional synaptic deficits, including reduced dendritic spine density, alterations in spine morphology, and diminished synaptic contact area, principally in the hippocampus and prefrontal cortex (Figure 2) [142,143]. Presynaptically, reductions in synaptophysin impair synaptic vesicle release and decrease excitatory neurotransmission, thus compromising activity-dependent plasticity such as long-term potentiation (LTP) [144,145]. Postsynaptically, age-related changes in PSD-95, a major scaffolding protein, destabilize postsynaptic densities and disrupt clustering of NMDA and AMPA receptors, causing reduced synaptic efficacy, impaired Ca^2+^ signaling, and reduced activation of downstream effectors that support learning and memory [146]. Ca^2+^ homeostasis is exacerbated in aging neurons [147], as dysregulation of VGCC and NMDA receptors [148,149], thereby producing inappropriate or insufficient Ca^2+^ transients. These disturbances impair activation of Ca^2+^-dependent pathways, such as calmodulin, CaMKII, and CREB, which are necessary for spine stabilization, synaptic consolidation, and activity-dependent gene expression [150,151,152]. Finally, in aging neurons, mitochondrial impairment dysregulates vesicle recycling, neurotransmitter reuptake, and ionic balance, while oxidative damage from ROS to synaptic proteins and membrane lipids further impairs synaptic stability and contributes to neuronal degeneration (Figure 2) [153,154].

Within the framework of glial cell biology, aged astrocytes show marked dysregulation of intracellular Ca^2+^ signaling, characterized by aberrant Ca^2+^ oscillations and reduced responsiveness to neurotransmitter-induced activation [126,155]. These perturbations in Ca^2+^ homeostasis impair the exocytotic release of synaptogenic molecules. Specifically, the secretion of extracellular matrix and synapse-promoting proteins like thrombospondins, and SPARCL1 are markedly downregulated in senescent astrocytes [78,156,157,158]. Thrombospondins promote the initial formation of excitatory synapses through interactions with neuronal α2δ-1 receptors [159], whereas SPARCL1 mediates the bridging of presynaptic neurexins and postsynaptic neuroligins to stabilize nascent synaptic contacts [160]. The cumulative effect of these astrocytic dysfunctions is a disrupted tripartite synapse environment, characterized by diminished astrocyte–neuron crosstalk, weakened synaptic adhesion, and impaired neurotransmitter clearance (Figure 2) [161,162,163].

Microglia maintain synaptic architectural homeostasis through dynamic modulation of synapse number and functional properties [164]. This regulation is organized through two mechanisms: (i) synaptic activity-mediated phagocytic elimination of superfluous or functionally compromised synaptic elements, commonly initiated through recognition of “eat-me” signals, including externalized phosphatidylserine and deposited complement components [165]; (ii) paracrine signaling via the secretion of cytokines, chemokines, and neurotrophic factors, which modulate synaptic plasticity, spine morphology, and neurotransmitter receptor trafficking [166]. During the aging process, microglial regulation of synaptic homeostasis becomes impaired, giving rise to maladaptive changes in neural circuit function [167]. Augmented sensitivity to “eat-me” signals increase the efficiency of activity-dependent phagocytic clearance, an excess further exacerbated by age-related alterations in complement pathway regulation (such as dysregulated C1q and C3 expression) which can result in excessive or aberrant synapse elimination in vulnerable neuronal populations [168]. Simultaneously, paracrine signaling is impaired, as aged microglia adopt a pro-inflammatory “primed” phenotype, characterized by increased secretion of pro-inflammatory cytokines (e.g., IL-1β, TNF-α, and IL-6), alongside diminished release of neurotrophic factors (e.g., BDNF) and other synaptogenesis-inducing molecules (e.g., TGF-β) [169,170]. This shift in the secretory profile fosters chronic low-grade neuroinflammation, disrupts dendritic spine integrity, and perturbs neurotransmitter receptor trafficking (particularly the composition of AMPA and NMDA receptor subunits), thus compromising synaptic plasticity (Figure 2) [171,172].

Finally, aging is linked to decline in OPCs, myelin fragmentation, and impaired metabolic coupling between axons and glial cells, specifically through reduced lactate shuttling through monocarboxylate transporter MCT1 and decreased expression of some myelin proteins (e.g., MBP and PLP1) [113]. These impairments disrupt the timing of synaptic input integration and compromise the fidelity of high-frequency action potential conduction, thereby indirectly contributing to synaptic dysfunction [173]. Furthermore, oligodendrocytes engage in bidirectional signaling with astrocytes and microglia to maintain extracellular K^+^ and glutamate homeostasis, regulate neurotrophic factor availability, and modulate local synaptic microenvironments [174,175]. Age-related oligodendrocyte loss disrupts these interactions, exacerbating excitotoxic stress, impairing Ca^2+^ buffering, and amplifying the detrimental impact of aging on synaptic structure (Figure 2) [55,176].

### 3.2. Myelin Degradation and White Matter Loss

Aging induces pronounced structural and functional reorganization of the CNS, with white matter being especially affected, mainly mediated by the regulatory and supportive activities of glial cells [41]. Oligodendrocytes, the myelinating glia of the CNS, are central to axonal insulation and rapid action potential conduction, yet they become increasingly vulnerable to age-related stressors [55]. Oligodendrocytes, the principal myelinating cells of the CNS, play a central role in white matter deterioration observed during aging [177]. With advancing age, oligodendrocytes exhibit both functional decline and structural vulnerability, leading to myelin degradation and impaired axonal support [178].

At the molecular level, mitochondrial dysfunction constitutes a critical driver of age-related myelin deterioration. The accumulation of mtDNA mutations, impairment of electron transport chain activity, and consequent reductions in ATP production compromise the energy-dependent processes essential for myelin maintenance, including the synthesis, trafficking, and integration of key myelin proteins like MBP, PLP1, and MAG [72,179]. Oxidative stress represents a key contributor, characterized by the accumulation of ROS and RNS in aging oligodendrocytes, resulting from mitochondrial dysfunction and a decline in intrinsic antioxidant capacity [72,180]. These reactive species induce lipid peroxidation of myelin sheaths, protein carbonylation, and DNA damage, triggering oligodendrocyte apoptosis through intrinsic mitochondrial pathways, involving Bcl-2 family regulation, Bax/Bak-dependent release of cytochrome c, and subsequent activation of caspases 3 and 9 [181,182].

On the other hand, the endoplasmic reticulum stress response is chronically activated in aged oligodendrocytes, culminating in the accumulation of misfolded myelin proteins [183,184]. Finally, aging compromises OPC differentiation [185], with disruptions in key signaling pathways (e.g., Wnt/β-catenin) impeding the maturation of OPCs into myelinating oligodendrocytes [186]. At the same time, reduced PI3K-Akt-mTOR signaling in aged oligodendrocytes compromises cell survival and suppresses lipid biosynthesis, a process essential for myelin membrane expansion [187,188].

### 3.3. Inflammaging: The Role of Chronic Low-Grade Inflammation in the Aging Process

Inflammaging, a chronic low-grade inflammatory state that progressively develops with advancing age, arises from a multifactorial interplay involving immune dysregulation, the accumulation of senescent cells, mitochondrial dysfunction, and defective resolution of inflammation [189]. Within the CNS, its impact is particularly pronounced, manifesting through the sustained activation of glial populations, most prominently microglia and astrocytes [190].

**Figure 3:**
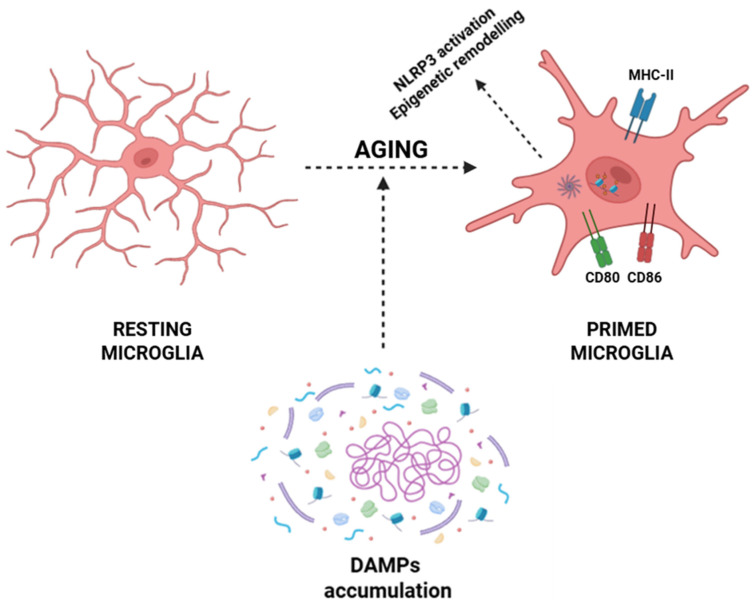
Aging-associated priming of microglia. Resting microglia exhibit a ramified morphology under homeostatic conditions. During aging, the accumulation of DAMPs, together with processes such as NLRP3 inflammasome activation and epigenetic remodeling, promotes the transition of microglia into a primed state. Primed microglia display altered morphology and upregulation of immune-related molecules, like MHC-II, CD80, and CD86, contributing to a heightened inflammatory responsivity. Abbreviations: DAMP (damage-associated molecular pattern), NLRP3 (NOD-, LRR- and pyrin domain-containing protein 3), MHC-II (major histocompatibility complex class II), CD80 (cluster of differentiation 80), and CD86 (cluster of differentiation 86).

Within the CNS, aging microglia undergo a phenotypic conversion toward a primed state characterized by exaggerated pro-inflammatory cytokine production (e.g., IL-1β, IL-6, and TNF-α), heightened expression of MHC-II and costimulatory molecules (CD80 and CD86), and sustained activation of pro-inflammatory transcriptional programs via NF-κB, AP-1, and IRF pathways [22,23,49]. This transition is driven by the progressive accumulation of DAMPs arising during inflammaging, including mitochondrial dysfunction with excessive production of ROS and release of oxidized mitochondrial DNA into the cytosol, which activates sensors such as cGAS and NLRP3 inflammasomes [191,192,193]. Aggregates of β-amyloid, hyperphosphorylated τ, and other misfolded proteins further activate the NLRP3 inflammasome, inducing its oligomerization, recruitment of the adaptor protein ASC, and caspase-1-mediated proteolytic processing of pro-IL-1β and pro-IL-18 into their functional cytokines [194,195,196,197,198,199]. Simultaneously, chronic exposure to low-grade inflammatory cues induces epigenetic remodeling that stabilizes microglia in a primed pro-inflammatory phenotype, thus sustaining a self-amplifying neuroinflammatory loop that contributes to age-associated CNS dysfunction and neurodegeneration (Figure 3) [111,112].

In aging, astrocytes acquire a reactive astrogliosis phenotype, characterized by upregulation of GFAP, activation of the JAK/STAT3 pathway, secretion of chemokines such as CCL2 and CXCL10, and release of complement components, including C3, which can opsonize synapses and govern their microglia-mediated elimination [17,18,46,78,200]. The aforementioned changes are further amplified by the dissemination of many SASP factors from senescent endothelial cells, OPCs, and peripheral immune cells infiltrating through a BBB rendered increasingly permeable by chronic cytokine exposure and oxidative stress [201,202]. SASP mediators are transcriptionally driven by DNA damage response signaling through ATM/ATR kinases, cGAS-STING-dependent NF-κB and STAT3 activation, and mTORC1-mediated translation, creating a paracrine loop that induces secondary senescence in glia and neurons [93,94,95,203,204].

Dysregulated synthesis of specialized pro-resolving lipid mediators (SPMs) such as resolvins, protectins, and maresins in aged glia impairs the termination of inflammatory responses, locking neural tissues into a state of chronic neuroinflammation that disrupts synaptic homeostasis, impairs neurogenesis in the hippocampal niche, accelerates myelin degradation by oligodendrocyte loss, and propagates several neurodegenerative cascades characteristic of Alzheimer’s, Parkinson’s, and other age-associated CNS diseases [205,206].

Finally, the inflammaging process is maintained through integrated feedback loops (DAMP accumulation, glial senescence, SASP secretion, mitochondrial ROS production, and inflammasome activation) that not only promote neuronal susceptibility and cognitive decline but also integrate systemic and brain-specific aging mechanisms into a unified pathological process, rendering glial cells both targets and amplifiers of the molecular circuitry that underlies age-related chronic inflammation [207].

## 4. Age-Related Disorders Driven by Glial Cell Dysfunction

Age-related neurodegenerative disorders are increasingly recognized as arising not only from neuronal pathology but also from the cumulative dysfunction of glial cells, which are critical for maintaining CNS homeostasis [208]. In Alzheimer’s disease, microglial cells adopt a persistently activated phenotype, characterized by increased expression of MHC-II molecules and pro-inflammatory cytokines, which exacerbate oxidative stress and neurotoxicity [209]. Dysfunctional microglia show reduced phagocytic activity and impaired lysosomal processing, which compromises the clearance of Aβ plaques and facilitates extracellular plaque deposition [210]. Simultaneously, astrocytes exhibit impaired glutamate uptake due to downregulation of excitatory amino acid transporters (EAAT1/2), metabolic insufficiency through decreased lactate shuttle support, and Ca^2+^ signaling dysregulation, collectively leading to synaptic dysfunction and dendritic spine loss [211]. Dysregulated astrocyte–microglia crosstalk further amplifies inflammatory signaling via the NF-κB and NLRP3 inflammasome pathways, accelerating neurodegeneration and cognitive decline [212].

In Parkinson’s disease, astrocytic and microglial dysregulation similarly contributes to dopaminergic neurons susceptibility in the substantia nigra [213]. Microglia adopt a reactive, pro-inflammatory phenotype in response to α-synuclein aggregates, releasing ROS and NO [214], while astrocytes fail to adequately buffer glutamate and detoxify ROS [215]. Furthermore, glial production of neurotrophic factors, like GDNF and BDNF, is reduced, decreasing neuronal survival signaling [216]. Some experimental models indicate that microglial-mediated inflammasome activation (via NLRP3) and impaired mitophagy synergistically accelerate dopaminergic cell death [217,218,219,220].

Age-related demyelinating pathologies, such as multiple sclerosis and age-associated white matter degeneration, are associated with oligodendrocyte dysfunction [221,222]. Senescent oligodendrocytes exhibit reduced myelin production and repair capacity, leading to axonal conduction deficits and increased risk of secondary degeneration [223]. Astrocytes contribute to the development of multiple sclerosis through the secretion of inhibitory extracellular matrix proteins, including chondroitin sulfate proteoglycans, which restrict remyelination, while microglial senescence reduces the clearance of myelin debris, further compromising regeneration [224].

Growing evidence also implicates glial dysfunction in vascular cognitive impairment and age-related cognitive decline, in which astrocytes and microglia fail to maintain neurovascular coupling, contributing to cerebral hypoperfusion, BBB breakdown, and synaptic loss [225,226]. In aging-associated affective disorders and later-life depression, glial senescence is increasingly recognized as a fundamental contributor [227]. Postmortem studies revealed reduced astrocyte density, altered astrocytic morphology, and microglial hyperactivation in regions such as the prefrontal cortex and hippocampus [228,229,230].

Finally, emerging evidence indicates a role for glial dysfunction in chronic pain syndromes and neuroimmune disorders [231]. Age-related microglial priming enhances sensitivity to peripheral injury, strengthening central sensitization pathways, while astrocytic dysregulation of cytokine and neurotransmitter release contributes to chronic pain states [232,233].

## 5. Promoting Glial Health: Therapeutic Options for Aging Brains

The integrity and functionality of glial cells are pivotal for maintaining neural homeostasis, synaptic plasticity, and metabolic maintenance of CNS function. During the aging process, glial cells demonstrate phenotypic and functional alterations, including reactive astrogliosis, microglial priming, and oligodendrocyte loss, which mutually contribute to neuroinflammation, impaired synaptic function, and cognitive decline. Therapeutic strategies aimed at preserving or restoring glial homeostasis are therefore critical for mitigating age-associated neurodegeneration and promoting healthy brain aging.

### 5.1. Lifestyle and Nutritional Interventions

Dietary interventions represent a potent modulatory axis for glial cell function, particularly in the context of aging. Caloric restriction (CR) and intermittent fasting (IF) have been extensively demonstrated to induce hormetic stress responses that confer neuroprotective benefits [234,235]. Mechanistically, these interventions accentuate autophagic flux, strengthen mitochondrial quality control, and mitigate oxidative stress in glial cells [236,237]. Beyond their systemic metabolic effects, CR and IF can modulate glial inflammatory states by suppressing pro-inflammatory cytokine production and promoting an anti-inflammatory or homeostatic glial phenotype [238,239]. Furthermore, nutrient composition critically influences glial health, as long-chain ω-3 polyunsaturated fatty acids (PUFAs), particularly docosahexaenoic acid (DHA) and eicosapentaenoic acid (EPA), incorporate into neuronal and glial membranes, enhancing membrane fluidity and receptor function [240]. These PUFAs have been demonstrated to reduce microglial activation and promote oligodendrocyte maturation and myelination [241]. On the other hand, dietary polyphenols (such as resveratrol, EGCG, and curcumin) show potent anti-inflammatory activities [242]. These compounds modulate ROS production, inhibit NLRP3 inflammasome activation, and modulate astrocytic and microglial senescence, thus mitigating age-associated neuroinflammation and supporting synaptic integrity [243,244,245].

On the other hand, diets enriched in bioactive compounds, such as polyphenols (e.g., resveratrol, epigallocatechin-3-gallate, and curcumin) and PUFAs exert significant effects on epigenetic regulation in glial cells [245,246]. However, the mechanisms underlying epigenetic regulation in glial cells remain understood and are currently the subject of ongoing research.

Regular physical exercise constitutes a potent, non-pharmacological strategy for preserving CNS homeostasis through modulation of glial function [247]. Aerobic exercise improves neurovascular coupling, boosts cerebral blood flow, and strengthens endothelial function, thus facilitating efficient delivery of O_2_ and nutrients to neurons and glial cells [248,249,250]. Exercise promotes the expression of several neurotrophic factors, most notably BDNF, which exerts pleiotropic effects on glial cells [251]. Enhanced BDNF signaling has been associated with a shift toward a neuroprotective microglial phenotype, reduced pro-inflammatory cytokine production, and strengthened astrocytic support of synaptic function [252,253]. Exercise also modulates astrocytic glycogen metabolism, increasing glycogen storage and mobilization, which supports sustained neuronal activity during periods of high energetic demand [254]. Moreover, physical activity stimulates oligodendrogenesis and myelin remodeling, which are essential for maintaining conduction velocity and network efficiency, mainly in aging brains [255,256,257]. Various animal studies have shown that voluntary wheel running and treadmill exercise strengthen myelin sheath thickness, promote the maturation of OPCs, and improve cognitive resilience under age-associated stress [258,259]. Ultimately, exercise-mediated promotion of antioxidant defenses in glial cells ameliorates oxidative damage and suppresses chronic neuroinflammatory signaling, thereby supporting the maintenance of CNS health [260].

### 5.2. Pharmacological Interventions

CNS aging is marked by sustained neuroinflammatory activity, disrupted metabolic regulation, and reduced neurotrophic support, all of which contribute to glial dysfunction and cognitive decline [261]. Pharmacological approaches are being explored to counteract these processes by targeting inflammation, trophic signaling, and metabolic homeostasis.

#### 5.2.1. Anti-Inflammatory Agents

Ibuprofen, a widely used NSAID, has been shown to counteract age-related inflammatory processes in the CNS by modulating glial cell activation [262]. Research in animal models indicates that chronic ibuprofen administration reduces reactive astrogliosis and microglial activation [263,264,265,266]. The reduction in glial reactivity correlates with decreased gliosis and improved cognitive outcomes, mainly in executive function [267,268]. Furthermore, mechanistic studies also reveal that ibuprofen influences the expression of NMDA receptor subunits and splice variants, with the most prominent effects evident in age-affected regions of the brain [267].

MCC950, a selective inhibitor of the NLRP3 inflammasome, has been shown to attenuate age-related neuroinflammatory responses through the modulation of glial cell activity [269]. Experimental data show that MCC950 inhibits microglial and astrocytic activation, thereby preventing microglial polarization toward the pro-inflammatory M1 phenotype [270]. This modulation exerts neuroprotective effects, evidenced by attenuated neuronal injury, demyelination, and oligodendrocyte loss [269]. In aged mice, MCC950 treatment has been linked to enhanced myelin integrity and increased densities of OPCs, supporting the notion that targeting NLRP3-mediated glial activation can relieve age-related and pathological alterations in the CNS [271].

#### 5.2.2. Neurotrophic Support

Exogenous delivery or endogenous enhancement of neurotrophic factors, including BDNF, GDNF, and IGF-1, has been identified as a promising therapeutic approach to mitigate age-associated impairments in glial cell functionality and to preserve neural homeostasis [272,273,274]. Mechanistically, BDNF predominantly engages the TrkB receptor, triggering downstream signaling cascades (like PI3K/Akt, MAPK/ERK, and PLCγ pathways) that converge to regulate glial cell survival, metabolic activity, and homeostatic function [275]. Exogenous administration of BDNF has been shown to influence aging glial cells by promoting the proliferation and maturation of oligodendrocytes, enhancing white matter repair and myelin integrity [276,277].

Similarly, IGF-1 interacts with the IGF-1R to enhance mitochondrial integrity, regulate lipid metabolism, and maintain proteostasis, processes that are markedly impaired in senescent astrocytes and oligodendrocytes [278,279]. Exogenous administration of IGF-1 exerts pronounced effects on aging glial cells, primarily by modulating neuroinflammatory responses and augmenting glial cell functional capacity [280,281]. Other studies show that IGF-1 acts as an anti-inflammatory agent by inhibiting astrocytic responses to inflammatory stimuli and by decreasing the pro-inflammatory M1 phenotype [282,283]. Moreover, IGF-1 supports oligodendrocyte survival and differentiation, helping to maintain myelin integrity in the aging CNS [284].

Finally, GDNF, via its interaction with GFRα1/RET receptor complexes, further promotes glial resilience by modulating redox homeostasis and providing trophic support to dopaminergic neural circuits [285]. Exogenous administration of GDNF in aged rodents constitutes a promising therapeutic strategy to ameliorate glial age-related dysfunction; however, further studies are required to validate this approach [286,287].

From another standpoint, small-molecule TrkB agonists and pharmacological modulators of IGF-1 signaling are under investigation as targeted regulators of glial survival, synaptic plasticity, and neurovascular interactions [288,289]. These interventions have the potential not only to attenuate age-related glial dysfunction and neuroinflammatory processes but also to promote healthy brain aging by preserving white matter integrity, cognitive resilience, and overall neural plasticity [288,289].

#### 5.2.3. Metabolic Modulators

Age-related mitochondrial dysfunction in glial cells is a crucial contributor to the disruption of cellular homeostasis during aging [14], and accordingly, pharmacological strategies targeting mitochondrial preservation have demonstrated promising efficacy in both preclinical models and early-phase clinical studies [290]. NAD+ precursors, including nicotinamide riboside (NR) and nicotinamide mononucleotide (NMN), strengthen intracellular NAD+ levels, thereby enhancing mitochondrial sirtuin activity, optimizing oxidative phosphorylation efficiency, and facilitating DNA repair processes [291,292,293]. In the same way, AMPK activators, including metformin, promote mitochondrial biogenesis, enhance fatty acid oxidation, and regulate cellular energy-sensing pathways, thus collectively restoring metabolic homeostasis in aged glial cells [294,295,296]. Finally, mitochondria-targeted agents, including MitoQ and SS-31, might be considered as potential therapeutic strategies to counteract the aging process in glial cells, given their protective effects against several neurodegenerative pathologies [297,298].

### 5.3. Emerging Biotechnological Approaches

Glial dysfunction is increasingly recognized as an essential driver of CNS aging, contributing to metabolic imbalance, impaired synaptic support, among others. To counteract these processes, several emerging biotechnological strategies are under investigation.

Adoptive transfer of glial progenitor cells seeks to replenish astrocytic and oligodendroglial populations, thus restoring trophic support and remyelination in aged circuits. In parallel, gene therapy and epigenetic modulation offer tools to enhance neuroprotective pathways or suppress pro-inflammatory programs within glial cells. Together with these approaches, nanotechnology-based delivery systems enable controlled delivery of therapeutic agents to glial populations, improving efficacy while minimizing side effects.

Together, these strategies represent a convergent effort to preserve CNS homeostasis and delay neurodegenerative progression by directly targeting the glial compartment.

#### 5.3.1. Adoptive Transfer of Glial Progenitor Cells

The implantation of glial progenitor cells, as well as astrocytes and oligodendrocytes derived from induced pluripotent stem cells (iPSCs), represents a highly promising therapeutic strategy for counteracting age-associated glial depletion [299,300]. These interventions aim not only to replenish depleted glial populations but also to restore essential metabolic, trophic, and homeostatic support to neurons, while regulating local neuroimmune responses in the aging CNS [301,302].

Mechanistically, engrafted astrocytes have been demonstrated to secrete an array of neurotrophic factors (e.g., BDNF, GDNF, and CNTF) that promote neuronal survival, dendritic arborization, and synaptic plasticity [303,304]. Moreover, engrafted astrocytes facilitate neuronal metabolic support by promoting lactate transport via monocarboxylate transporters (MCT1/4), regulating extracellular K^+^ and glutamate levels, and reinforcing antioxidant defenses [305,306]. Oligodendrocytes derived from iPSCs can facilitate remyelination through the expression of MBP and PLP, restoring saltatory conduction and improving axonal integrity in demyelinated or aging neural circuits [300,307].

In addition to providing trophic and metabolic support, transplanted glia modulates neuroinflammation, as engrafted astrocytes and oligodendrocytes communicate with microglia through cytokine and chemokine signaling (such as IL-10, TGF-β, and CX3CL1), thereby attenuating microglial hyperactivation and the secretion of pro-inflammatory mediators [304,308].

Preclinical studies further demonstrate that these transplanted glial populations are capable of integrating into existing neural circuits, forming functional gap junctions, and contributing to synaptic modulation as well as neurotransmitter recycling [309,310,311,312,313].

#### 5.3.2. Gene Therapy and Epigenetic Modulation

Targeted gene therapy aimed at modulating glial cell function represents a cutting-edge strategy for mitigating age-related neurodegenerative processes and preserving CNS homeostasis [314]. By enhancing the expression of several neuroprotective genes or selectively silencing pro-inflammatory and senescence-associated pathways within glial cells, gene therapy can immediately influence neuronal survival, synaptic function, and metabolic support [315]. For instance, viral vector-driven overexpression of neurotrophic factors like BDNF, GDNF, and IGF-1 in glial populations has been shown to improve synaptic plasticity, reduce oxidative stress, and enhance neuronal resilience in preclinical models of aging and neurodegeneration [316,317,318]. On the other hand, RNAi, CRISPR-Cas-mediated gene editing, or antisense oligonucleotides can be employed to downregulate the expression of pro-inflammatory cytokines, NLRP3 inflammasome components, and SASP mediators, thereby mitigating the chronic neuroinflammation that typifies the aged CNS [319,320,321].

Complementing gene therapy, epigenetic modulation provides a parallel and synergistic approach to restoring glial functionality [322]. Age-related transcriptional dysregulation in glial populations frequently results from changes in chromatin accessibility, alterations in histone post-translational modifications, and changes of DNA methylation patterns [323]. Histone deacetylase (HDAC) inhibitors, like compounds targeting HDACs (e.g., MS-275 and SAHA) can reverse transcriptional repression of neuroprotective genes, encouraging antioxidant defenses, anti-inflammatory signaling, and metabolic homeostasis [324].

#### 5.3.3. Nanotechnology and Drug Delivery Systems

Innovative nanocarrier platforms and BBB-penetrant delivery systems are emerging as transformative strategies for the modulation of glial cell activity within the aged CNS [325,326]. These methods harness the specific physicochemical properties of nanoparticles (such as size, surface charge, and functionalization with targeting ligands) to achieve cell-specific uptake by glial cells, thus enabling the localized delivery of neuroprotective compounds, anti-inflammatory agents, or gene-editing tools [327]. Surface modifications with antibodies (e.g., anti-TfR and anti-IR), other peptides (e.g., angiopep-2 and TAT), and/or small molecules allow nanoparticles to traverse the BBB via receptor-mediated transcytosis, circumventing the restrictive tight junctions that typically impede CNS drug delivery [328,329,330,331].

Mechanistically, nanocarriers can be engineered to release their cargo in a spatiotemporally controlled manner, responding to intracellular cues such as acidic pH within endosomes/lysosomes, elevated ROS, or specific enzymatic activity (e.g., matrix metalloproteinases or cathepsins), thereby strengthening intracellular bioavailability in target glial populations [332,333]. Following cellular uptake, these carriers release various small-molecule neuroprotective drugs that activate several antioxidant pathways (e.g., Nrf2, SOD2, and HO-1), which collectively counteract oxidative stress and mitochondrial dysfunction [334,335,336].

Moreover, advanced formulations such as liposomes, polymeric nanoparticles, dendrimers, and exosome-mimetic vesicles are being optimized for long circulation, minimal immunogenicity, and high BBB permeability [337,338,339,340]. Targeted delivery to glial cells also mitigates off-target effects and reduces systemic toxicity, which is critical when administering potent anti-inflammatory or gene-modifying agents in aged individuals [327].

## 6. Conclusions

The aging process exerts a profound and multifaceted impact on CNS integrity, with glial cells (astrocytes, microglia, and oligodendrocytes) emerging as critical mediators of age-related neural vulnerability. Emerging evidence indicates that these glial populations experience significant structural, functional, and molecular alterations during aging. Astrocytes show reduced metabolic support and impaired neurotransmitter recycling, compromising neuronal energy homeostasis and synaptic function. On the other hand, microglia adopt a pro-inflammatory and senescent phenotype, characterized by persistent low-grade activation, altered phagocytic capacity, and dysregulated pro-inflammatory cytokine production, which collectively propagate a neuroinflammatory milieu. Oligodendrocytes and their progenitor cells display diminished myelination efficiency and regenerative potential, contributing to deficits in axonal conduction and neuronal network stability.

The convergence of these glial abnormalities contributes to increased oxidative stress, disrupts synaptic plasticity, and undermines the capacity of the CNS to respond to injury or pathological insult. Notably, glial aging is not simply a passive outcome of chronological progression but represents some molecular and cellular adaptations, including modifications in gene expression, mitochondrial dysfunction, and disrupted intercellular signaling. This underscores the concept that glial cells are active determinants of CNS aging, rather than secondary responders to neuronal decline.

Therapeutically, targeting glial dysfunction represents a promising avenue to preserve CNS health and mitigate age-associated neurological disorders. Interventions aimed at restoring glial metabolic competence, modulating neuroinflammatory signaling pathways, promoting oligodendrocyte regeneration, or rejuvenating senescent glial populations hold substantial potential to enhance neural resilience. Moreover, the identification of robust biomarkers of glial senescence and dysfunction might enable early detection of CNS vulnerability and inform the development of precision therapeutics.

In summary, the interplay of aging and glial cell dysfunction constitutes a key determinant of CNS homeostasis, cognitive integrity, and susceptibility to neurodegenerative processes. Future research should prioritize a mechanistic understanding of glial senescence, explore interventional strategies that restore glial functionality, and integrate glia-centric approaches into broader frameworks for healthy brain aging. Such efforts are critical for translating fundamental insights into clinically meaningful strategies to promote longevity and neural health across the lifespan.

## Data Availability

Not applicable.

## Abbreviations

The following abbreviations are used in this manuscript:

Akt	Protein kinase B (PKB)
AMPA	α-Amino-3-hydroxy-5-methyl-4-isoxazolepropionic acid
AMPK	AMP-activated protein kinase
AP-1	Activator protein 1
ARF	Alternative reading frame protein
ASC	Apoptosis-associated speck-like protein containing a CARD
ATM	Ataxia-telangiectasia mutated
ATP	Adenosine triphosphate
ATR	ATM and Rad3-related
Bak	Bcl-2 homologous antagonist/killer
Bax	Bcl-2–associated X protein
BBB	Blood–brain barrier
Bcl-2	B-cell lymphoma 2
BDNF	Brain-derived neurotrophic factor
C3	Complement component 3
Ca^2+^	Calcium ion
CaMKII	Calcium/calmodulin-dependent protein kinase II
CCL2	Chemokine (C-C motif) ligand 2
CCL5	C-C motif chemokine ligand 5
CD80	Cluster of differentiation 80
CD86	Cluster of differentiation 86
CDK4/6	Cyclin-dependent kinases 4 and 6
cGAS	Cyclic GMP-AMP synthase
CHK1	Checkpoint kinase 1
CHK2	Checkpoint kinase 2
CMA	Chaperone-mediated autophagy
CNS	Central nervous system
CNTF	Ciliary neurotrophic factor
CR	Caloric restriction
CREB	cAMP response element-binding protein
CRISPR-Cas	Clustered regularly interspaced short palindromic repeats-CRISPR-associated protein
CX3CL1	C-X3-C motif chemokine ligand 1
Cx43	Connexin 43
CXCL10	C-X-C motif chemokine ligand 10
DAMP	Damage-associated molecular pattern
DDR	DNA damage response
DHA	Docosahexaenoic acid
DNA	Deoxyribonucleic acid
DNMT1	DNA methyltransferase 1
DSB	Double-strand break
EAAT1	Excitatory amino acid transporter 1
EAAT2	Excitatory amino acid transporter 2
ECM	Extracellular matrix
EGCG	Epigallocatechin-3-gallate
EPA	Eicosapentaenoic acid
ERK	Extracellular signal-regulated kinase
EV	Extracellular vesicle
FGF2	Fibroblast growth factor 2
GDNF	Glial cell line-derived neurotrophic factor
GFAP	Glial fibrillary acidic protein
GFRα1	GDNF family receptor alpha 1
H_2_O_2_	Hydrogen peroxide
H3K27ac	Histone 3 lysine 27 acetylation
H3K4me3	Histone 3 lysine 4 trimethylation
HDAC	Histone deacetylase
HDAC1	Histone deacetylase 1
HDAC2	Histone deacetylase 2
HDAC3	Histone deacetylase 3
HDAC8	Histone deacetylase 8
HO-1	Heme oxigenase 1
Hsp70	Heat shock protein 70
IF	Intermittent fasting
IFN-γ	Interferon gamma
IGF-1	Insulin-like growth factor 1
IGF-1R	Insulin-like growth factor 1 receptor
IL-10	Interleukin 10
IL-1β	Interleukin 1 beta
IL-6	Interleukin 6
IL-8	Interleukin 8
INK4a	Inhibitor of cyclin-dependent kinase 4a (p16 protein)
iPSCs	Induced pluripotent stem cells
IR	Insulin receptor
IRF	Interferon regulatory factor
JAK	Janus kinase
K^+^	Potassium ion
KFERQ	Lys-Phe-Glu-Arg-Gln peptide
LC3	Microtubule-associated protein 1A/1B-light chain 3
LTP	Long-term potentiation
M1	Pro-inflammatory microglial phenotype
MAG	Myelin-associated glycoprotein
MAPK	Mitogen-activated protein kinase
MBP	Myelin basic protein
MCT1	Monocarboxylate transporter 1
MCT1/4	Monocarboxylate transporter 1 and 4
MHC-II	Major histocompatibility complex class II
miRNA	MicroRNA
MMP-3	Matrix metalloproteinase 3
MMP-9	Matrix metalloproteinase 9
MS-275	N-(2-aminophenyl)-4-[N-(pyridine-3ylmethoxy-carbonyl)aminomethyl]benzamide
mtDNA	Mitochondrial DNA
mTOR	Mechanistic target of rapamycin
mTORC1	Mechanistic target of rapamycin complex 1
NAD+	Nicotinamide adenine dinucleotide
NADH	Nicotinamide adenine dinucleotide (reduced form)
NF-κB	Nuclear factor kappa-light-chain-enhancer of activated B cells
NLRP3	NOD-, LRR- and pyrin domain-containing protein 3
NMDA	N-methyl-D-aspartate
NMN	Nicotinamide mononucleotide
NO	Nitric oxide
NR	Nicotinamide riboside
Nrf2	Nuclear factor erythroid 2-related factor 2
NSAID	Non-steroidal anti-inflammatory drug
O_2_^−^	Superoxide anion
OPC	Oligodendrocyte precursor cell
OXPHOS	Oxidative phosphorylation
p16	Cyclin-dependent kinase inhibitor 2A (CDKN2A)
p21	Cyclin-dependent kinase inhibitor 1 (CDKN1A)
P2X7	Purinergic receptor P2X ligand-gated ion channel 7
P2Y12	Purinergic receptor P2Y, G-protein coupled, 12
p53	Tumor protein p53
PDGF-A	Platelet-derived growth factor A
PI3K	Phosphoinositide 3-kinase
PINK1	PTEN-induced kinase 1
PLCγ	Phospholipase C gamma
PLP1	Proteolipid protein 1
pro-IL-18	Pro-interleukin 18
pro-IL-1β	Pro-interleukin 1 beta
PRR	Pattern recognition receptor
PSD-95	Postsynaptic density protein 95
PUFA	Polyunsaturated fatty acid
Rb	Retinoblastoma protein
RET	Rearranged during transfection
RNAi	RNA interference
RNS	Reactive nitrogen species
ROS	Reactive oxygen species
SAHA	Suberoylanilide hydroxamic acid
SASP	Senescence-associated secretory phenotype
SOD2	Superoxide dismutase 2
SPARCL1	Secreted protein acidic and rich in cysteine-like 1
SPM	Specialized pro-resolving lipid mediator
SS-31	Elamipretide
STAT3	Signal transducer and activator of transcription 3
STING	Stimulator of interferon genes
TAT	Trans-activator of transcription
TfR	Transferrin receptor
TGF-β	Transforming growth factor beta
TLR4	Toll-like receptor 4
TNF-α	Tumor necrosis factor alpha
TrkB	Tropomyosin receptor kinase B
UPS	Ubiquitin–proteasome system
VGCC	Voltage-gated calcium channel
Wnt	Wingless/integrated
α2δ-1	Alpha-2-delta-1 subunit of VGCC
ω-3	Omega 3
